# Partnering with frail or seriously ill patients in research: a systematic review

**DOI:** 10.1186/s40900-020-00225-2

**Published:** 2020-09-11

**Authors:** Claire Ludwig, Ian D. Graham, Wendy Gifford, Josee Lavoie, Dawn Stacey

**Affiliations:** 1 University of Ottawa, Faculty of Health Sciences, School of Nursing, Roger Guindon Hall, 451 Smyth Road, Ottawa, Ontario K1H 8M5 Canada; 2 University of Ottawa, Faculty of Medicine, School of Epidemiology and Public Health, Ottawa, Ontario, Canada and Ottawa Hospital Research Institute, Clinical Epidemiology Program, 501 Smyth Road, Ottawa, Ontario K1H 8L6 Canada; 3 Geriatric Psychiatry Program, Royal Ottawa Mental Health Centre, 1145 Carling Avenue, Ottawa, Ontario K1Z 7K4 Canada; 4 Ottawa Hospital Research Institute, Clinical Epidemiology Program, 501 Smyth Road, Ottawa, Ontario K1H 8L6 Canada

**Keywords:** Patient engagement, Public patient involvement, Systematic review, Integrated knowledge translation, Co-production

## Abstract

**Background:**

The expectation to include patients as partners in research has steadily gained momentum. The vulnerability of frail and/or seriously ill patients provides additional complexity and may deter researchers from welcoming individuals from this patient population onto their teams. The aim was to synthesize the evidence on the engagement of frail and/or seriously ill patients as research partners across the research cycle.

**Methods:**

A systematic review was conducted using PRISMA guidelines. A search strategy included MEDLINE®, EMBASE®, Cumulative Index to Nursing and Allied Health Literature (CINAHL), and PsycINFO from database inception to April, 2019. Eligible studies were peer-reviewed qualitative, quantitative, and mixed methods research reporting on the engagement of frail and/or seriously ill patients as partners on research teams. The Mixed Methods Appraisal Tool was used to appraise study quality. Narrative analysis was conducted.

**Results:**

Of 8763 citations, 30 were included. Most studies included individuals with cancer on the research team (60%). Barriers included: lack of time and resources (50%), discontinuity in contribution (37%), and concerns for well-being (33%). Facilitators included: trust and mutual respect (60%), structural accessibility (57%), flexibility in timing and methods of engagement (43%), and attention to care and comfort, (33%). Perceived impacts for patients included: renewed personal sense of agency (37%) and emotional/peer support (37%). Impacts for researchers included sensitization to the lived experience of disease (57%) and an increased appreciation of the benefits of patient engagement (23%). Research design, execution, and outcomes, developed with patients, were deemed more suitable, relevant and reflective of patients’ priorities.

**Conclusions:**

There is emerging evidence to suggest that research partnerships with frail and/or seriously ill patients can be achieved successfully. Patients mostly report benefit from partnering with research teams. Frailty and/or serious illness do present legitimate concerns for their well-being but appear to be successfully mitigated when researchers ensure that the purpose of engagement is well-defined, the timing and methods of engagement are flexible, and the practical and emotional needs of patient partners are addressed throughout the process.

**Systematic review registration:**

The systematic review protocol was registered with the International Prospective Register of Systematic Reviews PROSPERO (CRD42019127994).

## Plain English summary

Patients are experts by experience and are becoming more active as partners on research teams. Patients who are frail and/or seriously ill do not appear to be engaged as research partners to the same extent as those living with more stable illness. The aim of this systematic review was to explore how frail and/or seriously ill patients have been engaged as partners in research.

In 30 studies, frail and/or seriously ill patients were engaged as research partners. They identified: research questions and outcomes important to patients; developed tools and processes more related to patients’ needs and experiences; helped collect and/or interpret findings; presented research results; and provided study oversight. Barriers to patients’ partnering were mostly related to concerns about their fragile health, their ability to process information and their likely limited ability to partner for the duration of the study due to declining health or death. When frail and/or seriously ill patients were engaged as partners in research, patients had a renewed sense of purpose and felt emotional support, research was more related to patients’ needs, and researchers gained greater insight into the lived experience of illness and suffering. Overall, it appears that frail and/or seriously ill patients can and should be included as research partners. Researchers can work to avoid unduly harming patient partners by being flexible and ensuring patients’ physical and emotional needs are addressed during the research process.

## Background

Over the past two decades, the commitment to engaging patients as partners in research has steadily gained momentum. International interest in patient engagement has been fostered by the belief that it can enhance the relevance, validity, and quality of research [1]. It is further postulated that research developed in this way will be more applicable to the needs of patients and hence more readily applied [2, 3]; thereby, legitimizing research that is often publicly funded [4, 5]. Patient engagement has become a moral and ethical imperative and, in some jurisdictions, particularly with marginalized communities, patient engagement also serves as a pre-requisite for research ethics approval [6–8]. The engagement of patients as partners in the design, execution and evaluation of health research is now an expectation of several principal funding programs [5, 9].

In Canada, as in many other countries, most major national and provincial research funding bodies promote engagement of patients throughout the entire process, from determining the research question to dissemination of the research results [10, 11]. A systematic review of 142 studies [2] established that, in most instances, it was possible for patients to contribute their expertise across the continuum of research; however, their engagement tended to be focused in the early stages of the study. The level of patient engagement in the process has varied in intensity and complexity depending on the nature of the research and information needs [11]. A more recent scoping review examining methods and outcomes of patient engagement confirmed that, in the absence of a validated framework, most efforts to engage patients continued to be limited to the early stages of engagement and did not appear to be maintained throughout the lifecycle of most research projects [12].

### Engaging patients as research partners

Patients are broadly defined as individuals with personal experience of a health condition [11]. There are numerous terms used for the concept of patient engagement in research including, but not limited to: ‘integrated knowledge translation’, ‘patient and public involvement’, ‘participation’, ‘patient engagement’, ‘public and patient engagement’ and ‘co-production’ [13, 14]. Patient engagement can be considered along a continuum from consultation at one end of the spectrum to partnership at the other end of the spectrum of engagement [15]. Research partnership is identified as patient membership on the research team, contributing to shared decision-making across the research process, engaged in the planning, execution and dissemination of research findings [15].

When partnering with patients, there is a shift from the researcher as sole expert to one where researchers and patients are both experts, working together to solve problems and co-generate knowledge [16]. Patients’ experiential knowledge (of illness) is not accessible to most researchers, but if leveraged appropriately, has the potential to complement researchers’ analytical skills and scientific perspective ([17] , p. 676). The concept of partnering with patients as equal team members has been demonstrated in clinical guideline development [18], by systematic review teams [19], and in the area of health and services improvement [20]. However, there are ongoing concerns about the need to balance rights to participation with efficiency and outcomes, [21, 22], particularly in disciplines that may lack the necessary infrastructure to support patient-facing activities (e.g., preclinical research) [23, 24].

### Partnering with frail and/or seriously ill patients

Inclusivity is an important principle in meaningful research partnerships with patients and places emphasis on equity of engagement in research [25, 26]. Whilst a number of reviews have concentrated on descriptions of the process and methods for various levels of patient engagement, little attention has been directed towards providing detailed accounts of patient characteristics [2, 12, 15, 27]. This oversight makes it difficult to gauge the inclusion of individuals from diverse patient populations.

The vulnerability of certain groups, such as frail and/or seriously ill patients (e.g., elderly patients with limited functional capacity, patients with high symptom burden, palliative patients), provides additional complexity to the engagement processes for prolonged and more intensive patient partnerships [27–29]. Frailty is classified as: a) geriatric condition involving functional decline, with increasing vulnerability to adverse events including mortality, morbidity, disability, hospitalization, and nursing home admission [30], or b) presence of multiple chronic conditions such as arthritis, heart failure, renal failure, and pulmonary disease leading to changes in functional ability [31], or c) presence of cognitive decline and dementia [30, 32]. Older adults living with frailty are a diverse group of patients that exhibit physical and/or cognitive impairments. Serious illness is defined as a condition that carries a high risk of mortality, negatively impacts quality of life and daily function, and/or is burdensome in symptoms or treatments [33]. Examples of serious illnesses are cancer (e.g., metastatic or hematologic), advanced liver disease, and advanced pulmonary diseases [33].

Patients who are frail and/or seriously ill have unique needs associated with symptoms related to their condition and/or treatment side effects which may offer researchers’ access to a lived experience of illness that is qualitatively different than those with more stable or chronic conditions [29]. Practical issues related to engaging frail and/or seriously ill patients as research partners appears to deter research teams from inviting, or even considering them for membership on the research team [28]. A recent scoping review exploring engagement of geriatric oncology patients found little evidence of patients’ inclusion as research partners [29]. Little is known about the engagement of frail and/or seriously ill patients as partners on research teams.

### Aim

The aim was to synthesize the evidence on the engagement of frail and/or seriously ill patients as research partners across the research cycle. The specific objectives were to: a) describe the contribution of frail and/or seriously ill patient partners to the stages of the research cycle (and associated research activities), b) identify the barriers and facilitators to partnering encountered by frail and/or seriously ill patients, and researchers, and c) describe the perceived positive and negative impacts of including frail and/or seriously ill patient partners in research from the perspective of patients, researchers, and the research itself.

## Methods/design

### Study design

A systematic review of qualitative, quantitative, and mixed methods studies was conducted with narrative synthesis. The Preferred Reporting Items for Systematic Reviews and Meta-Analyses (PRISMA) [34] guided the reporting. The study protocol was developed prior to the literature search and registered via PROSPERO (CRD42019127994).

### Guiding conceptual framework

The systematic review was guided by a conceptual framework comprised of two components (see Table 1). The first component utilizes a modified version of the Patient Service User Engagement in Research Framework originating from a prior systematic review by Shippee et al. [15], and addresses patient engagement at different stages of the research cycle and associated activities. The second component addresses the level of engagement in the decision-making process as defined by the International Association of Public Participation (IAP2) Spectrum of Public Participation (*see Supplementary file* *1**for additional detail*) [35]. The IAP2 spectrum denotes five levels of engagement (inform, consult, involve, collaborate, and empower) and has been used in Canada, Australia, New Zealand, Indonesia, Italy, Southern Africa and the USA to outline levels of engagement and promote best practices in patient and public engagement [36].

**Table 1:** Guiding conceptual framework for engaging frail and/or seriously ill patients in research

Stages of Research Cycle^**a**^	IAP2 Spectrum of Public Participation^b^
**Foundational phase** • Research priority setting – specific to disease, condition, or syndrome • Setting evidence-based patient engagement strategies – specific to disease, condition, or syndrome	**Inform** • Providing balanced and objective information to assist in understanding the problem, alternatives, opportunities and/or solutions
**Preparatory phase** • Agenda setting at the individual study level • Proposal development • Ethics application – including well-defined consent procedures • Acquiring funding/grant application	**Consult** • Seeking/obtaining feedback on analysis, alternatives and/or decisions
**Execution phase** • Study design & procedures • Recruitment strategies & tools • Data collection • Data analysis (reviewing & interpreting data)	**Involve** • Working directly with (patients) throughout the process to ensure concerns and aspirations are consistently understood and considered
**Translation phase** • Dissemination • Implementation • Evaluation	**Collaborate** • Partnering in each aspect of the decision (e.g., contributing to shared decision-making *across the research process)*
	**Empower** • Patients and members of the public provide final decision.

^a^Modified from Shippee et al. (2015) [15]

^b^Based on the IAP2 Spectrum of Public Participation (2014) [35]

### Data sources and search strategy

An electronic search strategy was developed with the assistance of an experienced health sciences librarian (KF) and adapted for the following databases: MEDLINE® (via Ovid), Cumulative Index to Nursing and Allied Health Literature (CINAHL via EBSCO), Excerpta Medica database (EMBASE® via Ovid), and PsycINFO® (via Ovid). The search strategy included a combination of key words and medical subject headings (MeSH) terms such as “patient engagement”, “patient involvement”, “patient-oriented research”. (*see Supplementary file* *2**for the complete Medline search strategy*). Reference lists of the included studies were manually reviewed to maximize the breadth of the review. There were no date limitations. The search strategy was executed from April 4, 2019 to April 6, 2019.

### Eligibility criteria

The population, intervention, control, outcomes, study design (PICOS) criteria were used to assess study eligibility [34] (see Table 2). All original studies of any design were eligible if they included frail and/or seriously ill patients as research partners at the level of involvement, collaboration, or empowerment throughout the research cycle (see Table 1). There were no language restrictions. In order to limit duplication, all systematic reviews were excluded after manually searching the reference lists of relevant reviews. Commentaries and editorials were excluded as well as studies that did not provide any details on patient perspectives or patient condition and when no full text was available.

**Table 2:** Study eligibility criteria: Modified (PICOS) Framework

PICOS [34]	Inclusion Criteria	Exclusion Criteria
**Participants (P)**	• Frail and/or seriously ill adult patients as per definitions for frailty and serious illness (e.g., elderly patients exhibiting physical and/or cognitive impairments, patients with high symptom burden due to acute illness or treatment effects, acute episodic illness, palliative patients; patients susceptible to adverse events including mortality, morbidity, disability, hospitalization, and nursing home admission).	• Studies where patients were excluded due to frailty of condition (physical and or cognitive) or deemed too ill to participate during acute episodes of serious illness or treatment. • Patients not identified as frail or seriously ill, i.e., survivors, chronic disease (focus on single disease without description of acuity/severity of condition). • Participants from broader community or public engagement (with no descriptors of frailty and serious illness) • Patients for whom there were no descriptors of physical characteristics or cognitive status. • Pediatric and youth patients (< 18 yrs).
**Phenomenon of Interest (I)**	• Engagement of frail and/or seriously ill patients as partners in research, i.e., at the level of involvement, collaboration, empowerment.	• Engagement of patients as objects of study, i.e., doing research *on* or *to.* • Engagement that took the form of informing patients of research activities, or at the level of consultation only.
**Comparator (C)**	No comparator	
**Outcome (O)**	• Methods and timing of engagement (i.e., stage(s) of research process). • Level of engagement. • Engagement strategies, factors associated with barriers and facilitators to engagement. • Positive and/or negative impacts of engagement on patient(s), researcher(s), research and/or ethical concerns.	• Primary research outcomes where patients were research participants only.
**Study Type (S)**	• Peer-reviewed qualitative, quantitative, or mixed methods studies.	• Letters. • Commentaries/editorials. • Studies reported in non-peer reviewed journals. • Conference abstracts/ presentations. • Dissertations. • Review articles.
**Language**	No language restrictions.	

### Study selection

Search results were uploaded to Covidence Systematic Review Software [37]. Following the removal of duplicates, citations were screened independently by two reviewers (CL, JL) based on title and abstract (level 1 screening) and full-text articles (level 2 screening). The studies were assessed against the inclusion and exclusion criteria. Full-texts that did not meet the eligibility criteria were excluded and the rationale was documented in the Covidence Systematic Review Software to facilitate ease of tracking and reporting.

### Data extraction

Data extraction forms were developed to provide a standardized and transparent method for examining the methodology and findings from the studies [38]. The forms were piloted on a subset of relevant papers that were included in the review and refined to ensure the extraction template met the specific objectives of the review. The following general characteristics were extracted: year of publication; title, aim, study design, country of conduct; number of frail and/or seriously ill patients engaged in research; patient condition with regard to reports of serious illness and/or frailty of patients. Engagement in research was extracted on four components: a) stages of the research cycle and associated activities within those stages; b) the level of engagement in the decision-making process, i.e., involvement, collaboration, and empowerment (see Table 1); c) barriers and facilitators to engaging frail and/or seriously ill patients in research; d) the described impacts of engaging frail and/or seriously ill patients. Data were extracted by two independent reviewers (CL, JL) and discrepancies resolved through discussion. A third party (DS) was available in the event that consensus could not be reached.

### Data analyses

Narrative descriptions were reported for all studies. Data were synthesized in accordance with the guiding conceptual framework, i.e., engagement during the research cycle and by level of engagement. No meta-analyses were conducted as the aim was to identify the scope and types of patient engagement. Additionally, the heterogeneity across studies regarding the design, patient populations, methods, measures used, and a lack of numeric outcomes reported inhibited meta-analyses.

### Quality assessment

Two independent reviewers (CL, JL) critically appraised included studies using the updated Mixed Methods Appraisal Tool (MMAT) [39]. The MMAT has been content validated, tested for inter-rater reliability and is increasingly utilized in the quality appraisal of systematic reviews of mixed studies [40–43]. Scores are based on criteria, which differ according to study type. Each study was appraised according to the criteria met and were ranked as having low, moderate, or high quality. But they were not excluded on the basis of low quality because the overall aim was to identify the scope and types of patient engagement. Reviewers resolved discrepancies through discussion and consensus.

## Results

### Search and selection results

**Fig. 1:**
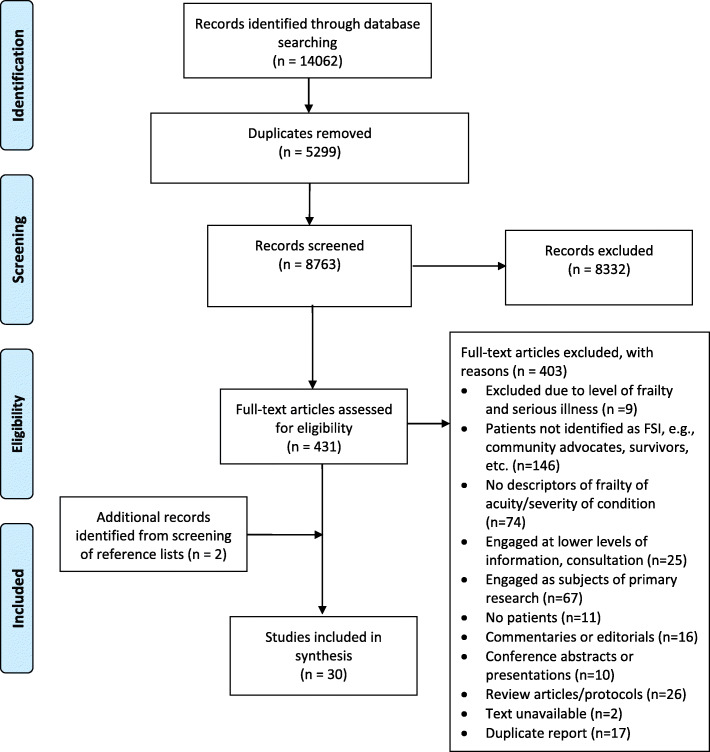
PRISMA Flow Diagram

**Fig. 2:**
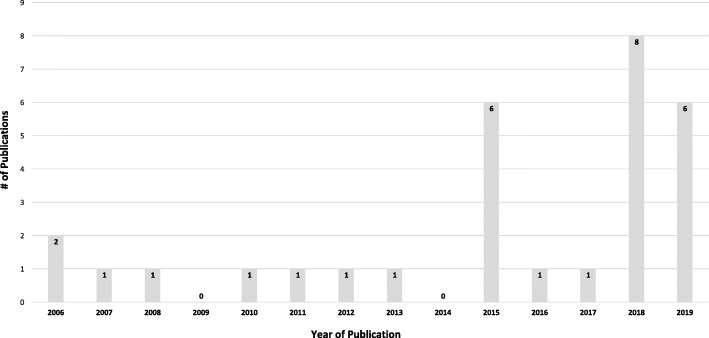
Number of publications by year (2006–2019)

There were 14,062 citations retrieved from electronic searching (see Fig. 1) [44]. After removing duplicates, 8763 original articles were screened, 431 full text reports were reviewed for eligibility, and 28 studies plus two additional studies identified through manual screening of reference lists in the included studies for a total of 30 studies met eligibility criteria. Included studies were published between 2006 and 2019, with a trend of increasing publications over time; 73% of studies were published within the last 5 years since 2014 (see Fig. 2).

### Characteristics of included studies

Of 30 studies, 20 used qualitative methods (67%), 2 used quantitative methods (7%), and 8 used mixed methods (27%) (see Table 3). All studies were published in English. Studies originated from: United Kingdom (*n* = 18 studies), Canada (*n* = 5), Denmark (*n* = 3), United States (*n* = 2), the Netherlands (*n* = 1), and Malawi (*n* = 1).

**Table 3:** Characteristics of included studies

Author, year, country of origin	Study objective related to this systematic review (from text)	Methodological approach and data collection	# FSI patients engaged
Absolom 2015 [45] UK	To provide an overview of how research collaborations with patient representatives have developed over time and how patient involvement has played a crucial role the success of local and national cancer research programs (eRapid study).	Qualitative; case study	14 patients on treatment, cancer survivors 2 additional patients on research S/C
Arain 2015 [46] UK	To explore different ways of involving consumers in cancer research in one regional network.	Quantitative; descriptive	15 patients on treatment, ex-patients, cancer survivors, caregivers
Bates 2018 [47] Malawi	To report on experiences and lessons learnt using Photovoice in Blantyre, Malawi to encourage its wider use in research and practice.	Qualitative; participatory action research (PAR)	6 patients with palliative care needs
Bethell 2018 [48] Canada	To engage persons with dementia, friends, family, caregivers, and health and social care providers to identify and prioritize their questions for research related to living with dementia and prevention, diagnosis, and treatment of dementia.	Mixed methods; James Lind Alliance Research Priority Setting Partnership (PSP) methods	7 persons with dementia 1 additional person with dementia on research S/C
Bethell 2019 [49] Canada	To engage people with lived or clinical experience of frailty and produce a list of research priorities related to care, support, and treatment of older adults living with frailty	Mixed methods; James Lind Alliance Research Priority Setting Partnership (PSP) methods	52 initial survey 6 interim prioritization # n/r for research S/C participation
Burns 2018 [50] US	To report outcomes of engaging patients and caregivers, identification of knowledge gaps, and prioritization of high impact research questions or recommendations related to hematopoietic cell transplantation.	Qualitative; focus groups	25 patients Patients also served on steering committee & working groups
Caldon 2010 [51] UK	To report on the process and consequences of consumer participation, rather than the findings of the illustrative (primary) research study	Qualitative; case study	2 patients
Chiu 2013 [7] Canada	To share the experience of engaging cancer patients/survivors in a participatory research study.	Mixed methods; participatory action research (PAR)	18 patients on treatment, ex-patients, cancer survivors
Collins 2015 [24] UK	To outline the challenges faced by the North Trent Cancer Research Network Consumer Research Panel model of Public & Patient Involvement.	Qualitative; case study	38 patients on treatment, ex-patients, cancer survivors
Corner 2007 [52] UK	To involve cancer patients across the UK in identifying priorities for research investment.	Qualitative; participatory action research & nominal group study	130 patients on treatment, ex-patients/cancer survivors
Cotterell 2011 [53] UK	To explore the personal impact of involvement on the lives of service users affected by cancer.	Qualitative; focus groups	64 patients on treatment, ex-patients/cancer survivors
Davis 2019 [54] UK	To consult frail older adults about services improvements and research topics associated with the design and delivery of discharge from hospital. To use successive PPIE processes to enable a permanent PPIE panel to be established.	Qualitative; focus groups and interviews	27 frail older adults
Froggatt 2015 [55] UK	To describe the experiences of people’s participation in patient and public involvement (PPI) in supportive and palliative care research.	Qualitative; semi-structured interviews	8 patients 1 patient on research S/C
Heaven 2016 [56] UK	To create a structure to enable meaningful, sustainable public involvement within the cmRCT framework.	Qualitative; case study	70 frail older adults
Iwata 2019 [57] US	To describe the benefits of patient-driven research in the field of head and neck oncology, review lessons learned from establishing partnerships with patients and caregivers and serve as a model for further patient-driven research endeavors.	Qualitative; case study	15 patients on treatment, ex-patients, cancer survivors
Jones 2017 [58] Canada	To identify research priorities in the management of kidney cancer.	Mixed methods; James Lind Alliance Research Priority Setting Partnership (PSP) methods	34 patients on treatment: 34 waiting surgery: 7 on research S/C (conflated with caregivers)
Jorgensen 2018 [59] Denmark	To report on the process of having current and former cancer patients involved as co-researchers.	Qualitative; case study	8 patients on treatment, ex-patients, cancer survivors
Jorgensen 2018 [60] Denmark	To investigate the impact of involving patient representatives as peer interviewers in a research project on patient empowerment.	Mixed methods; qualitative & quantitative analyses	16 patients on treatment, ex-patients, cancer survivors
Lechelt 2018 [61] Canada	To determine research priorities for patients with head and neck cancer.	Mixed methods (James Lind Alliance method for PSP)	104 patients on treatment, ex-patients, cancer survivors 5 patients on research S/C
Litherland 2018 [62] UK	To describe the involvement of people with dementia and carers as part of the IDEAL study	Qualitative; case study	3 persons with dementia
Littlechild 2015 [63] UK	To evaluate the impact of working with co-researchers from the perspective of multiple stakeholders on a project in which older people with dementia and older people from a black and minority ethnic community were involved as co-researchers.	Qualitative; case study	11 older persons with dementia and/or frailty
Parveen 2018 [64] UK	To report the process of involving a diverse range of experts-by-experience approach within the Caregiving HOPE study, and its impact on research processes and outcomes.	Qualitative; case study	1 older person with dementia
Perkins 2008 [65] UK	To determine patients’ priorities for palliative care research through a questionnaire study	Quantitative; survey	19 patients 10 patients piloted tool
Piil 2019 [66] Denmark	To identify future research agendas that reflect the concerns and unexplored areas of interest for patients with life-threatening cancer, their relatives and the clinical specialists during the cancer trajectory.	Qualitative; focus groups	6 patients 2 patients on research S/C
Schölvinck 2019 [67] The Netherlands	To identify and prioritize research needs of hematological cancer patients and people who have undergone a stem cell transplantation.	Mixed methods; focus groups, interviews, questionnaire	19 patients interviewed 27 patients in focus group 146 patients surveyed 3 patients on research S/C
Stephens 2015 [68] UK	To identify top 10 research priorities relating to mesothelioma, and identify those unanswered questions that involved an intervention, in order to aid translation into answerable research questions.	Mixed methods; James Lind Alliance Research Priority Setting Partnership (PSP) methods	168 patients surveyed 6 patients at consensus meeting
Stevenson 2019 [69] UK	To involve individuals with dementia as co-researchers in analysis of research findings to enhance validity through a process of applying multiple perspectives to data analysis.	Qualitative; case study	4 persons with dementia
Tanner 2012 [70] UK	To report on the process of involving older people with dementia in all stages of the research process.	Qualitative; case study	3 persons with dementia
Wright 2006 [71] UK	To provide detail of collaborative participation of patients and carers in the design and conduct of participatory research study in setting the cancer research agenda.	Qualitative; participatory approach	22 patients & caregivers
Wright 2006 [72] UK	To describe the experiences of involving palliative care patients as co-researchers in end of life research.	Qualitative; case study	15 patients

S/C = research steering committee

The number of patients in the studies ranged from one [64] to 168 [68] with a median of 16 patients. There were 11 (37%) studies where patients were engaged as a group with caregivers and/or other stakeholders (e.g., ex-patients, survivors, patient representatives/ advocates, or members of the public) [7, 24, 46, 52, 53, 57–61, 72].

### Characteristics of patients in included studies

Of 30 studies, 18 (60%) included patients with specific cancer diseases: 10 heterogeneous cancers, 2 blood cancers, 2 head and neck cancers, 2 breast cancer, one kidney cancer, and one mesothelioma (see Table 4). Other studies included patients/persons with dementia (*n* = 6), older adults with frailty (*n* = 3), and palliative patients including malignant and non-malignant disease (*n* = 3). Patient characteristics of frailty and/or serious illness were mostly reported in relation to: receipt of active treatment(s) associated with high symptom burden (*n* = 18), receipt of palliative or end-of-life care (*n* = 3), higher levels of cognitive impairment (*n* = 6), and physical frailty associated with old age (*n* = 3).

**Table 4:** Patient characteristics and partnering activities

Study	Disease or diagnosis	Age (years)	Ethnicity or cultural identity	Description of illness severity acuity/frailty (from text)	Highest level of engagement	Research activities where patients provided input (from text)	Stages of the research cycle			
							Foundation	Preparation	Execution	Translation
Absolom 2015 [45]	Heterogeneous cancers - including gastro-intestinal, breast, prostate, and gynecological	50–70 yrs	n/r	Patients on active treatment for cancer.	Collaborate	Grant writing; proposal development; research design; recruitment strategy development; tool refinement; implementation & dissemination. *2 patients on the steering committee (SC) which oversaw and advised the study.*		√	√	√
Arain 2015 [46]	Heterogeneous cancers	n/r	“diversity”	Patients on treatment for cancer type; including colorectal, breast, lung, brain and prostate.	Collaborate	Grant writing; proposal development; research design; tool refinement (patient information sheets for clinical trials, questionnaires); advice for increasing trial recruitment, conducting patient interviews. Patients also sat on project team.		√	√	√
Bates 2018 [47]	Heterogeneous cancers	n/r	n/r	Patients receiving palliative care for advanced cancer.	Collaborate	Engaged in data collection and data analysis, dissemination activities.		√	√	√
Bethell 2018 [48]	Dementia	n/r	n/r	Different types/stages of dementia – varying degrees of cognitive impairment.	Collaborate	Identification and prioritization of research questions. *1 person with dementia included on the steering committee which oversaw and advised the study. Persons with dementia were involved in: promoting surveys and recruitment.*	√	√	√	
Bethell 2019 [49]	Older Adults with Frailty	n/r	n/r	Those with lived experience of frailty.	Collaborate	Identification of research priorities. *People with lived experience of frailty included on steering committee which oversaw and advised the study.*	√	√	√	
Burns 2018 [50]	Hematological malignancies	n/r	n/r	Patients who have undergone hematopoietic cell transplant.	Collaborate	Identified research priorities. Provided advice on patient engagement. Patients also participated on SC and working groups throughout the entire research cycle (details and outcomes of contribution provided).	√	√	√	√
Caldon 2010 [51]	Breast cancer	n/r	n/r	Patients with cancer. One patient partner died prior to publication of the study.	Collaborate	Co-development of the project – tools, documentation, and processes. Also involved in dissemination and co-authorship.		√	√	√
Chiu 2013 [7]	Breast cancer	n/r	n/r	Some participants on active treatment.	Collaborate	Provided input through all phases of the research from grant development to dissemination of study findings. Other activities included refinement of research questions, survey development, data analysis, presentations, and co-authorship.		√	√	√
Collins 2015 [24]	Heterogeneous cancers & palliative	22–75	n/r	Level of acuity not documented in cancer patients but includes palliative patients	Collaborate	Co-researchers across different projects from influencing the research agenda through to dissemination as co-authors and presenters at conferences.		√	√	√
Corner 2007 [52]	Heterogeneous cancers (including breast, gastrointestinal, lung, hematological, etc.) & palliative	30–70	n/r	16% on active treatment; 13% receiving palliative care. *Inclusion of other stakeholders,* e.g.*, caregivers, ex-patients (cancer survivors).* *Patients were excluded if deemed by clinical team to be too unwell, have complicating health factors or liable to be distressed by participating.*	Collaborate	Identification of research priorities. The co-researchers ‘co-owned’ the study with the unit, and as such had a direct influence on all aspects of the study, including data collection, analysis and dissemination of study findings.	√	√	√	√
Cotterell 2011 [53]	Heterogeneous cancers, COPD, Stills Disease, Parkinson’s Disease	41–78	“diversity”	Patients receiving active treatment and patients receiving palliative care (for non-malignancies). *Inclusion of other stakeholders,* e.g.*, caregivers, ex-patients (cancer survivors)*	Collaborate	Involved as integral members of the research team throughout the length of the study; data collection, analysis and dissemination of study findings.		√	√	√
Davis 2019 [54]	Frail older adults	n/r	Pakistani, Somalian, Yemeni	Frail older adults.	Collaborate	Identification of research topics. Provided advice on methods of patient engagement to develop sustainable infrastructure. Developed a PPI structure. *Patients/caregivers included on steering committee.*	√	√	√	
Froggatt 2015 [55]	Heterogeneous cancers	51–84	n/r	Patients experiencing recurrence of disease and those receiving ongoing treatment	Collaborate	Research partners across different studies in cancer research collaborative. Provided input regarding barriers to patient engagement. The term research partner was proposed by the co-applicant patient representative on the management group as reflecting the nature of the PPI working that was to be developed in the collaborative	√	√	√	
Heaven 2016 [56]	Frailty	75+	n/r	Older adults with frailty.	Collaborate	Engaged throughout a number of studies from grant writing/proposal development, research conduct, dissemination. Participation on research steering/advisory committees.		√	√	√
Iwata 2019 [57]	Head and neck cancers	35–74	10% Asian, Hispanic or Latino	Included patients on active treatment. *Inclusion of other stakeholders,* e.g.*, caregivers, ex-patients (cancer survivors).*	Collaborate	Engaged in identification of research priorities, hypothesis generation, feedback on tools and processes, clinical flow and dissemination.	√	√	√	√
Jones 2017 [58]	Kidney cancer	n/r	n/r	Included patients on current active treatment and those awaiting surgical treatment.	Collaborate	Identifying and prioritizing research questions. *7 Patients/caregivers included on steering committee; contributed throughout study design and execution; defining the scope of the partnership, development of the protocol, identifying potential partners and stakeholders, and oversight of the process*.	√	√	√	
Jorgensen 2018 [59]	Heterogeneous cancers	n/r	n/r	Included patients on active treatment. *Inclusion of other stakeholders,* e.g.*, a caregiver, ex-patients (cancer survivors).*	Collaborate	Engaged throughout research cycle: co-application on grants, literature review participation, outcome and tool development, feedback on the conduct of the research, presentations, co-authorship.		√	√	√
Jorgensen 2018 [60]	Heterogeneous cancers	n/r	n/r	Included peer interviewer with advanced age and stage of illness. *Co-researchers also included caregivers, ex-patients (cancer survivors).*	Collaborate	Involved in study design, conduct of research (conducting peer interviews), data analysis.		√	√	
Lechelt 2018 [61]	Head and neck cancers	n/r	n/r	Broad spectrum of patients, varying tumor types and sites, including newly diagnosed, those on current active treatment. *Inclusion of other stakeholders,* e.g.*, caregivers, ex-patients (cancer survivors).*	Collaborate	Identification of research priorities. *5 patients on the steering committee which established consensus on desired scope and inclusion/exclusion criteria for the project regarding: respondent groups, question categories; tumor types/site; developed the survey; oversaw all aspects of the project.*	√	√	√	
Litherland 2018 [62]	Dementia	n/r	n/r	Different types/stages of dementia – varying degrees of cognitive impairment.	Collaborate	Engaged in shaping project materials, providing feedback on questionnaires and interview processes, reviewing emerging theoretical themes, and presenting project findings.		√	√	√
Littlechild 2015 [63]	Dementia	n/r	“diversity”	Older persons with varying types/stages of dementia – varying degrees of cognitive impairment.	Collaborate	Engaged at all stages of the study, including: designing the research method and tools, identifying key themes and findings at the analysis stage, dissemination activities.		√	√	√
Parveen 2018 [64]	Dementia	n/r	n/r	Different types/stages of dementia – varying degrees of cognitive impairment	Collaborate	Engaged in discussing study progress, findings and interpretation of data		√	√	√
Perkins 2008 [65]	Heterogeneous cancers	65 median	n/r	Included palliative patients with a prognosis of 6 months or less.	Involve	Patient input into identification of research domains, piloting of questionnaires prior to prioritization of research questions.	^a^	√	√	
Piil 2019 [66]	Primary malignant brain tumor and acute leukemia	22–59	n/r	Life threatening cancer diagnosis, characterized by poor and uncertain prognosis, undergoing aggressive and intensive oncological treatments resulting in a complex symptom burden.	Collaborate	Identifying and prioritizing research questions. *Patients included on steering committee and contributed throughout study design & execution; defining scope of the partnership, development of the protocol, identifying potential partners and stakeholders, and oversight of the process. Additional details included in published study protocol* [73]*.*	√	√	√	√
Schölvinck 2019 [67]	Hematological malignancies	19–75+	n/r	Patients from all disease phases and types.	Collaborate	Identification and prioritization of research questions and outcomes. *Patient representatives included on the research steering/advisory committee.*	√	√	√	
Stephens 2015 [68]	Mesothelioma	n/r	n/r	Patients with high symptom burden.	Collaborate	Identification and prioritization of research questions. *Patients sat on the research advisory committee which oversaw and advised the study. .*	√	√	√	
Stevenson 2019 [69]	Dementia	< 65–75+	n/r	Cognitive impairment - early to mid-stage dementia.	Involve	Engaged in deriving meaning from the data, identifying and connecting themes.			√	
Tanner 2012 [70]	Dementia	60–77	n/r	Cognitive impairment – progressive during the study.	Involve	Engaged as co-researchers involved in conducting interviews.			√	
Wright 2006 [71]	Heterogeneous cancers	n/r	n/r	Includes patients undergoing treatment. *Inclusion of other stakeholders,* e.g.*, caregivers, ex-patients (cancer survivors).*	Involve	Engaged in the design and conduct of the study (including co-facilitation of focus groups). Also engaged in subsequent data analysis and dissemination activities.		√	√	√
Wright 2006 [72]	Disease/s not specified (palliative)	n/r	n/r	Patients receiving palliative care.	Collaborate	Engaged in the design and conduct of the study. Co-research role throughout the course of the study.		√	√	√
						Totals: n (%)	13 43%	28 93%	30 100%	18 60%

*n/r* not reported

^a^Research prioritization reported in prior publication [74]

Of 30 studies, 12 (40%) reported the ages of the patient-partners [24, 45, 52, 55–57, 61, 65–67, 69, 70] and six (6/12) of them also included patients 75 years of age and above [24, 55, 56, 67, 69, 70]. Five of 30 studies (17%) reported on ethnicity [46, 53, 54, 57, 63]; three (3/5) of which reported ethnicity more broadly in terms of “diversity” [46, 53, 63].

### Patient partner research roles: research stages and activities

No studies reported engagement of frail and/or seriously ill patients at the level of empowerment, i.e., the provision of primary direction and governance to a given research endeavor. The highest level of engagement was reported in four of 30 studies (13%) where collaboration was demonstrated across all four stages of the research cycle (see Table 4). Patients in these studies partnered in activities including, but not limited to: delineation of the scope of the partnership, contribution to study design, co-leadership on working groups during study execution, data analysis, dissemination activities, and adoption of decision-making roles on research steering/advisory committees [50, 52, 57, 66].

Seven studies (23%) included patients in research priority setting at the broader level of biomedical specialty/disease/condition, rather than at the individual study level [48, 49, 54, 58, 61, 67, 68]. These studies included patients who were representative of the condition as partners on research steering/advisory committees and who contributed to shared decision-making across the research study cycle.

Eleven studies (37%) described collaboration with frail and/or seriously ill patients across the latter three stages of the research cycle (preparation, execution, and translation) at the individual study level [7, 45, 47, 51, 53, 59, 62–64, 71, 72]. Patients partnered in a variety of different activities that mostly included: assistance with grant applications, input into study design, co-design of project materials, recruitment strategies, data analysis, dissemination activities, and decision-making at research steering/advisory committees. Four (13%) studies included frail and/or seriously ill patient partners from research collaboratives or networks who assisted with the identification of appropriate patient engagement strategies specific to frail and/or seriously ill populations at a broader system level. Patients described contributing to grant writing, proposal development, tool refinement, conducting interviews, representing research findings, and co-authorship on papers across different studies [24, 46, 55, 56].

Four studies (13%) described patient roles during key stages of the research process, rather than across the research cycle [60, 65, 69, 70]. Patients participated in activities at the execution stage of the research cycle, where they piloted research tools, served as peer interviewers, assisted in other forms of data collection, or interpreted data sets [60, 65, 69, 70].

### Barriers and facilitators to partnering with patients

#### System level factors

**Fig. 3:**
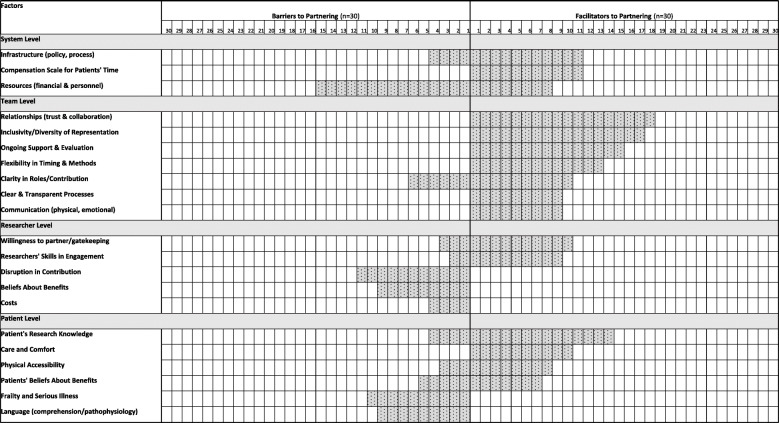
Themes and sub-themes of barriers and facilitators to partnering with frail and/or seriously ill patients

The most commonly cited barrier for researchers to partner in research with frail and/or seriously ill patients was resource constraints, including financial concerns, human resource capacity for support, and the time commitment required for meaningful engagement (15/30) [7, 24, 45–47, 54, 56, 59, 60, 62–64, 69, 71, 72] (see Fig. 3). Researchers also cited lack of formal infrastructure and policy, poorly defined governance mechanisms, and inconsistent processes to support meaningful patient partnerships as a system level barrier (4/30) [24, 53, 59, 64].

Both patients and researchers reported the need to establish consistent, formal compensation frameworks in order to recognize patient contribution and reimburse patients for their time, travel, and incidental costs (11/30) [7, 45–47, 56, 59, 61–64, 66]. Researchers’ stressed the importance of having a rigorous macro and micro level infrastructure with appropriate policy and governance mechanisms to support successful and meaningful patient partnership beyond a singular study (11/30) [24, 46, 48, 53, 54, 56, 57, 59, 64, 69, 72]. Relatedly, studies made direct reference to the significance of ensuring that funding for patient engagement is integrated into the research structure in order to facilitate and sustain patient engagement activities (8/30) [24, 45, 46, 54, 59, 62, 64, 69].

#### Team level factors

Lack of role clarity and expectations related to the contribution of patients throughout the research cycle was cited as a barrier to meaningful engagement by both patients and researchers (6/30) (see Fig. 3) [24, 45, 53, 59, 63, 69].

The most commonly cited facilitator to meaningful engagement with frail and/or seriously ill patients as partners in research was to establish a collaborative team environment built on trust, mutual respect, and openness (18/30) [7, 24, 45, 47, 51, 53, 57, 59, 60, 62–64, 66, 67, 69–72]. Researchers also stressed the importance of promoting structural accessibility as a facilitator to meaningful engagement, with an emphasis on inclusivity and diversity of representation (i.e., ensuring that patient partners were representative of varied ethnocultural and socioeconomic groups) (17/30) [7, 24, 45, 47–49, 52, 53, 56, 57, 59, 62–64, 68, 71, 72]. The importance of regular contact, ongoing support, feedback, and team de-briefing was recognized as a requirement to effective partnership for both patients and researchers (15/30) [7, 24, 45, 47, 51, 53, 54, 57, 59, 60, 62, 64, 67, 70, 72]. Flexibility in the timing, methods and modes of contribution (13/30) [7, 45, 50, 53, 54, 59, 62–65, 69, 70, 72], clarity in roles and the expected contribution of patients throughout the partnership (10/30) [24, 45, 46, 51, 53, 55, 57, 59, 60, 69], and clear and transparent processes for all members of the team (9/30) [24, 50–52, 59, 60, 63, 64, 70] were cited by both patients and researchers as key facilitators to the process. Facilitating communication through provision of multiple mechanisms for input and feedback, and limiting overly technocratic jargon was also perceived as vital to enabling patients’ contribution (9/30) [7, 24, 45, 51, 55, 57, 61, 68, 70].

#### Researcher level factors

The most commonly cited perceived barrier of researchers to partnering with frail and/or seriously ill patients in research was related to their concerns about patients’ potential lack of continuity in contributions throughout the research cycle due to deterioration in patients’ health or cognition, or death (11/30) (see Fig. 3) [7, 45, 47, 53, 55, 57, 59, 61, 68, 70, 72]. The second most common barrier was researchers’ uncertainty about the value or overall benefit of patient engagement, particularly given the outcomes of the partnership on research may not be visible for some time (9/30) [24, 46, 52, 53, 56, 57, 62, 69, 72]. Other researcher barriers were perception that research outputs identified by patient partners may not be fully aligned with the initial objectives of the project or might be too costly to implement (4/30) [24, 60, 63, 67], concern for placing additional or perceived unnecessary burden on patients (3/30) [47, 52, 65], and lack of familiarity and confidence in patient engagement, particularly where patients assume a partnership role (2/30) [56, 62].

Facilitators were researchers’ willingness to share decision-making with patients as essential to partnering with patients (10/30) [7, 24, 48, 50, 51, 53, 56, 59–61]. Another facilitator was researchers’ knowledge and expertise of patient engagement practices as vital to mitigating potential harms of engagement(9/30) [7, 24, 47, 51, 54, 57, 59, 62, 66].

#### Patient level factors

The most common patient level barrier was being frail and/or experiencing severe illness or limited cognitive status (10/30) [47–49, 52, 65, 66, 68, 70–72] (see Fig. 3). The second most common barrier was communication difficulties due to diminished capacity for comprehension, heightened emotional distress due to subject matter material, or pathophysiology (9/30) [24, 48, 55–57, 65, 67, 68, 70]. Other barriers were patients’ apprehension about the impact of their engagement and their capacity to influence action and outcomes of the research process (5/30) [53, 55, 57, 62, 68], perceived reservations about the extent to which patient partners possess the requisite knowledge and skills for research (4/30) [24, 60, 63, 69], and limited accessibility and concerns related to patients’ potential difficulty to physically attend meetings (3/30) [7, 54, 57].

Skills building by providing basic training for patients in research methods and research ethics, was cited by both patients and researchers as a key facilitator to building confidence in contribution and partnership (14/30) [7, 45–47, 51, 56, 57, 59, 60, 63, 69–72]. Another facilitator for engagement was the provision of practical and emotional support, and comfort (e.g., refreshments, quiet spaces) (10/30) [7, 47, 53, 57, 61, 62, 64, 69, 70, 72]. Other facilitators for patients were ensuring physical accessibility to meeting spaces (8/30) [7, 47, 54, 62, 64, 69, 70, 72], and patients’ altruistic beliefs that their involvement would improve care and outcomes for others (7/30) [45, 51, 53, 54, 57, 59, 63].

### Impacts

#### Perceived impact on patients

The most commonly cited positive impact to partnering in research was described by patients as a renewed sense of personal agency in the face of debilitating disease and loss of self-esteem (11/30) [7, 45, 47, 51, 53–55, 62, 63, 69, 70] (see Table 5). Patients also described positive impacts stemming from relationships formed with other patients and members of the research team which appeared to provide additional emotional support in their illness journey (11/30) [7, 51, 53, 55, 57, 62–64, 66, 69, 70]. Patient partnership was cited as having a positive beneficial impact for patients in relation to incorporation of their priorities for research questions and meaningful outcomes (10/30) [7, 24, 50, 51, 54, 56, 57, 61, 65, 67]. The development of new skills and knowledge (8/30) [45, 51, 55, 59, 60, 62, 63, 71] and acquisition of knowledge about their own disease/condition were also perceived by patients to be positive personal impacts (3/30) [51, 55, 69].

**Table 5:** Impacts of Patient Engagement (*N* = 30 studies)

**Patient Level -Perceived Impacts**	
**Positive Impacts**	**Negative Impacts**
11 (37%) Renewed sense of purpose/agency [7, 45, 47, 51, 53–55, 62, 63, 69, 70]	
11 (37%) Emotional/peer support [7, 51, 53, 55, 57, 62–64, 66, 69, 70]	5 (17%) Emotional vulnerability or emotional distress [7, 47, 55, 71, 72]
10 (33%) Incorporation of patients’ priorities for research and outcomes [7, 24, 50, 51, 54, 56, 57, 61, 65, 67]	
8 (27%) Develop new knowledge and skills [45, 51, 55, 59, 60, 62, 63, 71]	5 (17%) Physical/cognitive fatigue [7, 47, 53, 55, 72]
3 (10%) Acquire insights into disease and treatment [51, 55, 69]	
**Researcher – Perceived Impacts**	
**Positive Impacts**	**Negative Impacts**
17 (57%) Sensitizes researchers to experiential knowledge not gained at the bench or the bedside. Recognizing human experience [7, 45, 47, 50–52, 54, 55, 57, 59, 62, 63, 66, 67, 69, 70, 72]	
7 (23%) Challenges negative/ambiguous beliefs and perceptions of utility of patient partnerships [52, 59, 62, 63, 65, 71, 72]	
4 (13%) Increase interpersonal skills and highlighted significance of partnerships in research [51, 59, 62, 63]	
	15(50%) Investment and expenditure of time and resources [7, 24, 45–47, 54, 56, 59, 60, 62–64, 69–71]
	2 (7%) Complexity/intensity of the process may serve as an impediment to meeting project timeline [7, 64]
**Research Level - Perceived Impacts**	
**Positive Impacts**	**Negative Impacts**
13 (43%) Improves/informs research design, execution, and translation [7, 51, 54–56, 59, 62–64, 69–72]	
13 (43%) Research tools (e.g., consent and data collection form), processes (e.g., recruitment and retention), and methods are more relevant [7, 45–47, 51, 56, 57, 59, 62–64, 70, 71]	
11 (37%) Outcomes are identified as being more relevant to patients [46, 50, 51, 54, 63, 64, 66, 69–72]	
11 (33%) Patients’ input offers directions for researchers and research funding agencies – generation of new ideas [24, 45, 48, 49, 51, 52, 57, 61, 65, 67, 68]	
9 (30%) Research outputs are more accessible to the public [24, 45, 47, 51, 52, 56, 57, 64, 69]	
6 (20%) Research priorities ranked by patients reflect applicability to the lived experience of illness, frailty, and/or treatment [24, 48, 49, 52, 58, 61]	
2 (7%) Democratization of allocation of research resources [49, 52]	
1 (3%) Increased transparency and accountability for publicly-funded research [55]	

Perceived negative impacts for patients were cited as potential physical and/or cognitive fatigue related to the effort required during engagement (5/30) [7, 47, 53, 55, 72]. Increased emotional vulnerability and the potential for distress in reliving their illness and related negative experiences were also cited as perceived negative impacts to patient as partners (5/30) [7, 47, 55, 71, 72].

#### Perceived impacts on researchers

Perceived positive impacts of partnering with frail and/or seriously ill patients in the research process were cited as increasing researchers’ awareness, and sensitizing them to the lived experience of illness and suffering (17/30) [7, 45, 47, 50–52, 54, 55, 57, 59, 62, 63, 66, 67, 69, 70, 72]. Partnering with patients was reported to challenge negative or ambiguous views held by researchers about the utility of patient engagement (7/30) [52, 59, 62, 63, 65, 71, 72]. The potential to enhance interpersonal skills and promote inter-disciplinary collaboration (4/30) were also cited as positive impacts to researchers engaging frail and/or seriously ill patients as partners in research [51, 59, 62, 63].

The negative impacts described by researchers engaging frail and/or seriously ill patients as partners in research were described in relation to the potential strain on scarce resources (particularly related to funding and the human resource capacity required to support patient engagement activities) (15/30) [7, 24, 45–47, 54, 56, 59, 60, 62–64, 69–71]. The additional complexity of the process and time required for engaging patient partners was also cited as a potential impediment to advancing project objectives and meeting timelines closely aligned with research funding cycles (2/30) [7, 64].

#### Perceived impact on the research

Researchers partnering with frail and/or seriously ill patients cited positive impacts on the research itself, with the design, execution and end of grant translation of research perceived as more applicable to those populations for whom the research is intended to serve (13/30) [7, 51, 54–56, 59, 62–64, 69–72]. On a more tangible level, including patients in the research process was also described as having a positive impact on the development of research tools (e.g., consent and data collection tools), processes (e.g., recruitment and retention), and methods that were more appropriate for use with frail and/or seriously ill patients (13/30) [7, 45–47, 51, 56, 57, 59, 62–64, 70, 71]. Research produced with patient partners was also perceived to incorporate outcomes more relevant for frail and/or seriously ill populations (11/30) [46, 50, 51, 54, 63, 64, 66, 69–72], and generated new ideas and direction for researchers and funders (11/30) [24, 45, 48, 49, 51, 52, 57, 61, 65, 67, 68]. Research produced with patients is also perceived to produce outputs that are more accessible to patients (9/30) [24, 45, 47, 51, 52, 56, 57, 64, 69], was more reflective of the lived experience of illness, frailty, and/or treatment impacts (6/30) [24, 48, 49, 52, 58, 61]; facilitated democratization of the allocation of scarce funds (2/30) [49, 52], and increased transparency and accountability for public funds (1/30) [55].

### Study quality

All 30 studies provided evidence of relevant sources of data appropriate for the research question and used a research design relevant to address the research question. Of the 20 qualitative studies (66%) in the review, most were rated as high quality using the MMAT [47, 50, 52, 53, 55, 57, 59, 63, 66, 69–72]. Seven (7/20) of the qualitative studies were rated moderately lower because it was difficult to determine whether interpretation of the results was sufficiently substantiated by data [24, 45, 51, 54, 56, 62, 64]. For the two quantitative studies (6.7%) [46, 65], there was a risk of nonresponse bias in both studies, particularly in one study where those deemed too ill were excluded from the opportunity to participate [65]. For the eight mixed methods studies (26.7%) [7, 48, 49, 58, 60, 61, 67, 68], five had risk of nonresponse bias [48, 49, 58, 61, 67] and three reported interpretation of the qualitative results that was not sufficiently substantiated by the data [48, 61, 68] (see Table 6).

**Table 6:**
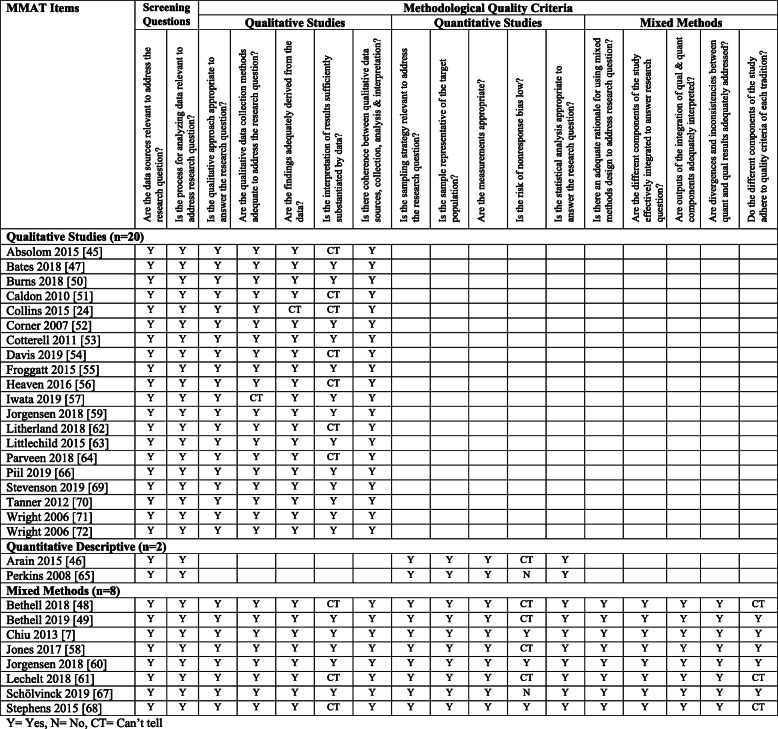
Quality appraisal results using MMAT [39]

*Y* Yes, *N* No, *CT* Can’t tell

## Discussion

The overall aim of this review was to synthesize the evidence on the engagement of frail and/or seriously ill patients as research partners across the research cycle. The 30 studies included in the review provide an indication of an upward trend in the inclusion of frail and/or seriously ill patients as partners in research over the past decade, with a marked increase in the number of studies in the past 5 years. Most studies included patients with cancer, with fewer studies partnered with patients who had dementia and/or frailty, or patients with palliative care needs. There was evidence of research partnerships with frail and/or seriously ill patients across the research cycle. These activities engaged patients on research related to setting priorities, selecting outcomes considered important to patients, grant review, tool development, research conduct, and dissemination of findings. These findings lead to the following three key points for discussion.

### Barriers, facilitators and impacts to engaging frail and/or seriously ill patient in research

The barriers and facilitators to partnering with frail and/or seriously ill patients (e.g., funding, infrastructure, role clarity, capacity building for both patients and researchers, structural inclusivity, trust and willingness to collaborate) are similar to those reported in other systematic and scoping reviews of patient engagement [2, 12, 22, 28, 75–77]. When engaging frail and/or seriously ill patients as partners across the research cycle, the degree of illness and/or frailty, and potential instability in patients’ health warrants more concern for wellbeing, but it should not serve to prevent initial or ongoing engagement [47]. Patients with high symptom burden and/or at end-of-life have expressed willingness and capacity for engagement in the development, conduct and dissemination of research [47, 65, 66, 72, 75, 76]. However, it is essential to confront researchers’, clinicians and caregivers’ concerns about over-burdening already frail and/or sick patients so that active and passive gatekeeping to engagement is minimized. Patients should be provided with the opportunity to accept or refuse opportunities to be engaged in research partnership in a manner that minimizes potential harm to them. The emphasis on how research partnership can and should be achieved is crucial in addressing the reservations that teams have in engaging frail and/or seriously ill patients beyond the level of consultation only.

Both patients and researchers should work to ensure clarity in patients’ roles and their expected contribution throughout the study so that their input is not perceived as tokenistic [45, 46, 57]. Unintended symbolic or inauthentic gestures with frail and/or seriously ill patients assumes a greater level of magnitude, particularly when quality of life is already compromised or life-span may be limited. Providing flexibility in the timing and methods for frail and/or seriously ill patients to contribute to the research process is critical to enabling partnerships given fluctuations in health and/or cognition [53, 54, 62]. Research teams have discussed the need for flexibility by engaging different patients who are representative of the frail and/or seriously ill population at different points and for different tasks during the project, such as design and grant writing, tool development, peer interviews, and dissemination [7, 45, 49, 52]. Enabling partnership with frail and/or seriously ill patients requires research teams to pay extra attention to the care and comfort of their patient partners, (e.g., providing refreshments, assisting with the logistics of attending meetings, ensuring comfortable and quiet rooms, and regular touch points) [7, 45, 47, 53, 57, 62, 64, 70]. The need to provide practical and emotional support has also been recognized in recent scoping reviews of patient and caregiver engagement in dementia research and palliative care research [28, 76].

There is ongoing deliberation about the paucity of evaluation of patient engagement in research, especially the long-term impacts related to research implementation and ongoing use of research findings [12, 22, 76, 78]. Interestingly, no reports of negative impacts on the research itself were found in the studies included in this review, which may reflect a bias in over-reporting positive impacts of patient engagement, or may suggest that evaluation efforts are more focused on short and intermediate term impacts of partnering with patients [79]. Insufficient evaluation and poor reporting of the negative impacts of patient engagement are described elsewhere in the literature and point to lack of methods and rigorous evaluation tools [22, 80]. Without validated evaluation frameworks and consistent identification of both positive and negative outcomes, there is a risk that anecdotal accounts, and perceived barriers to partnership will dominate the discourse of engagement and undermine the successes [79, 81].

Given the population of focus, it was surprising to have identified few negative impacts on patients. Negative outcomes were primarily defined as potential depletion of physical and emotional resources, and the likelihood of experiencing emotional distress through reliving painful illness experiences, exposure to undesirable information, or experiencing the direct suffering of others [45, 47]. It is difficult to establish whether the limited number of negative impacts identified is due to lack of evaluation or lack of reporting [12, 22, 28]. However, while a limited number of negative impacts were cited, the possible magnitude of these impacts should not be under-estimated and every effort is required by research teams to mitigate these potentially deleterious impacts. Similarly, when examining the potential impact on researchers partnering with frail and/or seriously ill patients, it is interesting to note that researchers described exposure and sensitization to the lived experience of illness and suffering, yet failed to acknowledge the concomitant emotional labor and associated burden that invariably comes with exposure to suffering [63, 82]. Issues of loss and grief are readily acknowledged for patient-partners following a decline in health or the death of others on the team [7]. However, it would appear that feelings of grief and loss, and the subsequent impact to emotional well-being, is not as readily acknowledged for researchers [82]. Failure to address these issues may leave many researchers ill-prepared to deal with emotionally demanding and difficult situations, cause unintended harm, and serve as a deterrent for both patients and researchers alike.

Evaluating the impact of partnering with frail and/or seriously ill patients is essential; limited evidence suggests that patients experienced several positive impacts, particularly when more intensive levels of engagement occurred. The potential emotional benefits described by patient partners (e.g., a renewed sense of purpose whilst coping with a disease over which they have little control, and/or the emotional support from peers on the research team) may in fact serve as a protective factor against emotional distress and vulnerability, and may also serve to quell researchers’ hesitation in partnering with them [7, 45, 47, 51, 64].

### Discontinuity of contribution

Consistent and predictable contribution is an important consideration for teams embarking on a partnership with patients, more so for those involving frail and/or seriously ill patients on research teams. Concern for well-being is critical and is cited as a barrier to both initial and ongoing engagement. Discontinuity of contribution is a commonly anticipated barrier to engaging those most frail and/or ill (i.e., patients receiving palliative care, those with progressive dementia, or experiencing aggressive disease progression) [53, 70]. Patients’ contribution will be lost or interrupted most often due to deterioration in their health or death, and it is incumbent on researchers to mitigate this. Paradoxically, discontinuity of contribution is rarely acknowledged when related to an improvement in condition, and yet, with advances in treatment approaches, particularly within oncology, many serious illnesses beyond the acute treatment phase are now considered chronic conditions [83]. If the purpose of including frail and/or seriously ill patients as partners in research is to provide access to the lived experience of their illness and leverage that knowledge to shape the research that is produced, the concept of discontinuity of contribution needs to be expanded to include situations when patient partners move from serious illness into remission, cure, or survivorship. The transition from serious illness to a period of more stable illness undoubtedly shifts the perspective and lived experience of patients. As such, it may be argued that over time they become less able to speak to the immediate lived experience of serious illness and more acute suffering. As patients are invited to participate in all stages of the research process, it is important to ensure patients within various stages of the illness trajectory are provided with equal opportunity to partner in the very research that is intended to benefit them [84].

### Weighing up the costs of partnership

There are moral, ethical, and practical reasons to engage frail and/or seriously ill patients as partners in research [85]; but researchers need to consider whether the impact or benefits of their engagement is warranted by the supplementary costs they will inevitably incur [79]. Facilitators for partnering with frail and/or seriously ill patients will invariably involve additional investments of time, money, and human resources to compensate for the accompanying administrative and emotional burden that research teams undertake in the endeavor [2, 28, 77]. Appropriate funding must be made available to teams dedicated to engaging frail and/or seriously ill patients as research partners, particularly when factoring in the need to address patients’ emotional and physical needs throughout the course of engagement [7, 47]. Therefore, it is necessary to optimize efforts at patient engagement to ensure expertise of patients who truly represent illness across the trajectory, particularly with regard to frailty and/or serious illness.

#### Strengths and limitations

The diversity of nomenclature describing patient engagement combined with a deficiency of standardized reporting and lack of specific indexing may have resulted in some relevant studies being undetected [2, 15]. There are distinctions between what constitutes a “patient,” “service user,” or member of the “public,” which pose additional methodological challenges for identification, recruitment and reporting [86]. Moreover, trajectories of disease progression, acute episodic exacerbation, and aggressive treatment regimens create challenges for defining frail and/or seriously ill patients [87]. To mitigate the challenges generated by issues of nomenclature and the potential fluidity of patients’ condition, the search strategy was designed intentionally to be broad in order to cast a wide net for potentially relevant papers.

Further effort was taken to review the reference lists of the included studies and recently published reviews on patient engagement. To mitigate potential bias two independent reviewers were involved during study screening, data extraction, and critical appraisal. The reviewers met numerous times throughout the review process to discuss and remain consistent. All supporting files were reviewed, attention was paid to descriptors of patient condition, and associated published study protocols, where available, were traced and reviewed. Of particular relevance, one of the reviewers was a patient who was representative of being seriously ill, experiencing illness and high treatment burden at the time of the review. The second reviewer works in a direct clinical role with frail and vulnerable populations. Co-authors have clinical expertise in oncology, palliative care, frail elderly care, integrated knowledge translation, systematic review methods, and community based participatory research and were instrumental in further addressing clinical and methodological issues during the review. There were few studies reporting on the engagement of frail and/or seriously ill patients. Hence, in the spirit of transparency and inclusion, none of the lower quality studies were excluded. Interestingly, quality issues in the quantitative studies were related to the potential for non-response bias whereby those deemed too ill were not engaged as research partners.

## Conclusion

Engaging frail and/or seriously ill patients as research partners has offered research teams a unique insight into understanding what it is like to live with a debilitating and fragile condition to develop research that more accurately addresses their needs. This review provides limited, but promising evidence that it is possible to successfully engage frail and/or seriously ill patients as partners in research without causing them harm. However, researchers need to ensure the purpose of engagement is well-defined, the timing and methods of inclusion are flexible, and the practical and emotional needs of patient partners are addressed. This review also highlights the need for more rigorous reporting of patient characteristics alongside the experiences, benefits, harms and impacts of their engagement in order to build best practices for engaging this vulnerable population.

## Supplementary information

**Additional file 1: Supplementary File 1.** International Association of Public Participation (IAP2) Spectrum of Public Participation.

**Additional file 2: Supplementary File 2.** Medline Search Terms.

**Additional file 3: Supplementary File 3.** PRISMA 2009 Checklist for systematic review.

## Data Availability

Not applicable.

## Abbreviations

CINAHL: Cumulative Index to Nursing and Allied Health Literature
EMBASE: Excerpta Medica Database
IAP2: International Association of Public Participation
MEDLINE: Medical Literature Analysis and Retrieval System Online
MMAT: Mixed Methods Appraisal Tool
PCORI: Patient-Centered Outcomes Research Institute
PICOS: Population/participants, Interventions, Comparators, Outcomes, and Study designs
PRISMA-P: Preferred Reporting Items for Systematic Reviews and Meta-Analyses Protocols
PROSPERO: Prospective Register of Systematic Reviews
PsycINFO: Psychological Information Database
