# Supplementary figures and images for: Partnering with frail or seriously ill patients in research: a systematic review
Source: Res Involv Engagem. 2020 Sep 11;6:52. doi: 10.1186/s40900-020-00225-2 (PMC7488581; doi:10.1186/s40900-020-00225-2)

**Supplementary File 3**


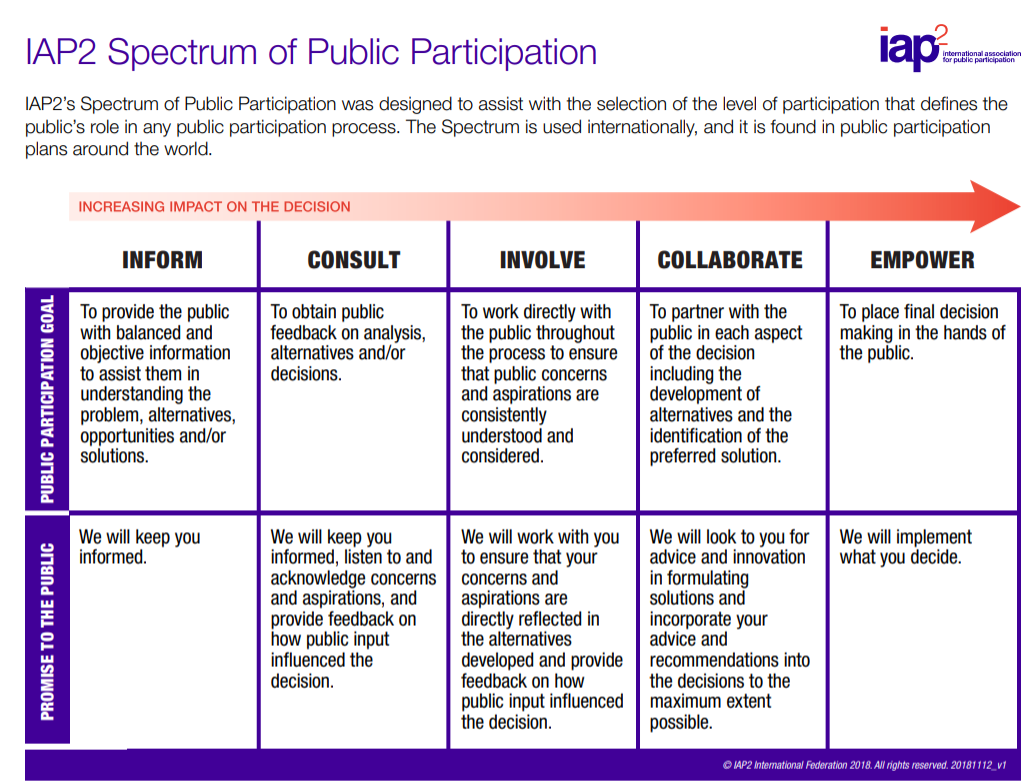


Reproduced with permission of IAP2 International Federation.

Supplement: Supplementary file 1 — Additional file 1: Supplementary File 1. International Association of Public Participation (IAP2) Spectrum of Public Participation. [file 40900_2020_225_MOESM1_ESM.docx]
