# Supplementary material for: Partnering with frail or seriously ill patients in research: a systematic review
Source: Res Involv Engagem. 2020 Sep 11;6:52. doi: 10.1186/s40900-020-00225-2 (PMC7488581; doi:10.1186/s40900-020-00225-2)
Supplement: Supplementary file 2 — Additional file 2: Supplementary File 2. Medline Search Terms. [file 40900_2020_225_MOESM2_ESM.docx]

**Supplementary File 2**

Medline Search Terms

| Steps | Query |
| --- | --- |
| #1 | Patient Participation/ |
| #2 | (patient* adj2 participat*).tw. |
| #3 | (patient* adj2 engag*).tw. |
| #4 | (patient* adj2 research*).tw. |
| #5 | (patient* adj2 empower*).tw. |
| #6 | (patient* adj2 involv*).tw. |
| #7 | (patient* adj2 collabor*).tw. |
| #8 | (patient* adj2 partner*).tw. |
| #9 | (patient* adj2 consult*).tw. |
| #10 | (consumer* adj2 participat*).tw. |
| #11 | (consumer* adj2 engag*).tw. |
| #12 | (consumer* adj2 research*).tw. |
| #13 | (consumer* adj2 empower*).tw. |
| #14 | (consumer* adj2 involv*).tw. |
| #15 | (consumer* adj2 collabor*).tw. |
| #16 | (consumer* adj2 partner*).tw. |
| #17 | (consumer* adj2 consult*).tw. |
| #18 | (user* adj2 participat*).tw. |
| #19 | (user* adj2 engag*).tw. |
| #20 | (user* adj2 research*).tw. |
| #21 | (user* adj2 empower*).tw. |
| #22 | (user* adj2 involv*).tw. |
| #23 | (user* adj2 collabor*).tw. |
| #24 | (user* adj2 partner*).tw. |
| #25 | (user* adj2 consult*).tw. |
| #26 | (stakeholder* adj2 participat*).tw. |
| #27 | (stakeholder* adj2 engag*).tw. |
| #28 | (stakeholder* adj2 research*).tw. |
| #29 | (stakeholder* adj2 empower*).tw. |
| #30 | (stakeholder* adj2 involv*).tw. |
| #31 | (stakeholder* adj2 collabor*).tw. |
| #32 | (stakeholder* adj2 partner*).tw. |
| #33 | (stakeholder* adj2 consult*).tw. |
| #34 | (citizen* adj2 participat*).tw. |
| #35 | (citizen* adj2 engag*).tw. |
| #36 | (citizen* adj2 research*).tw. |
| #37 | (citizen* adj2 empower*).tw. |
| #38 | (citizen* adj2 involv*).tw. |
| #39 | (citizen* adj2 collabor*).tw. |
| #40 | (citizen* adj2 partner*).tw. |
| #41 | (citizen* adj2 consult*).tw. |
| #42 | (lay* adj2 participat*).tw. |
| #43 | (lay* adj2 engag*).tw |
| #44 | (lay* adj2 research*).tw. |
| #45 | (lay* adj2 empower*).tw. |
| #46 | (lay* adj2 involv*).tw. |
| #47 | (lay* adj2 collabor*).tw. |
| #48 | (lay* adj2 partner*).tw. |
| #49 | (lay* adj2 consult*).tw. |
| **#50** | 1 or 2 or 3 or 4 or 5 or 6 or 7 or 8 or 9 or 10 or 11 or 12 or 13 or 14 or 15 or 16 or 17 or 18 or 19 or 20 or 21 or 22 or 23 or 24 or 25 or 26 or 27 or 28 or 29 or 30 or 31 or 32 or 33 or 34 or 35 or 36 or 37 or 38 or 39 or 40 or 41 or 42 or 43 or 44 or 45 or 46 or 47 or 48 or 49 |
| #51 | (translational adj3 research*).tw. |
| #52 | (shared adj3 learn*).tw. |
| #53 | exp Evidence-Based Practice/ |
| #54 | evidence-based practice.tw. |
| #55 | Biomedical research/ |
| #56 | Health Services Research/ |
| #57 | Clinical Trial/ |
| #58 | delphi*.tw. |
| #59 | (research adj utilization).tw. |
| #60 | (research adj develop*).tw. |
| #61 | (research adj implement*).tw. |
| #62 | (research adj translat*).tw. |
| #63 | (guideline* adj implement*).tw. |
| #64 | (guideline* adj develop*).tw. |
| #65 | (knowledge adj2 exchange).tw. |
| #66 | (knowledge adj2 translation).tw. |
| #67 | 51 or 52 or 53 or 54 or 55 or 56 or 57 or 58 or 59 or 60 or 61 or 62 or 63 or 64 or 65 or 66 |
| **#68** | **50 and 67** |
| #69 | Limit 68 to humans |
